# Recurrent Giant Ovarian Cysts in Biological Sisters: 2 Case Reports and Literature Review—Giant Ovarian Cysts in 2 Sisters

**DOI:** 10.3390/healthcare13060656

**Published:** 2025-03-17

**Authors:** Shuaibin Liu, Qianru Zeng, Lina Hu, Biao Zeng, Yi Wu, Chenxi Wang, Min Zhou, Xiaoling Gan

**Affiliations:** 1 Department of Obstetrics and Gynecology, The Second Affiliated Hospital of Chongqing Medical University, No. 288, Tianwen Avenue, Chayuan, Chongqing 400010, China; 304395@hospital.cqmu.edu.cn (S.L.); zengqianru@hospital.cqmu.edu.cn (Q.Z.); 302502@hospital.cqmu.edu.cn (L.H.); cqzb1018@sina.com (B.Z.); cqmuwuyi@163.com (Y.W.); 2 Fujian Key Laboratory of Innate Immune Biology, Biomedical Research Center of South China, College of Life Science, Fujian Normal University, Fuzhou 350117, China; wangchenxi.cy@outlook.com

**Keywords:** gynecological neoplasia, ovarian cysts, sisters, surgery

## Abstract

**Background:** Ovarian follicular cysts often resolve spontaneously, with giant forms being a rarity. Cases of giant ovarian follicular cysts in biological sisters without clear familial predisposition are even exceptional. **Cases Presentation:** Two biological sisters presented to our hospital with large pelvic masses in the setting of a clinical and biological hyperandrogenism. After surgical removal, pathology confirmed the diagnosis of ovarian follicular cysts. Recurrence was detected shortly after surgery, with both sisters displaying similar clinical courses. Chromosomal screening showed no abnormalities. Hormonal analysis revealed elevated anti-Müllerian hormone (AMH), prolactin (PRL), and testosterone, alongside low FSH and LH levels. Family exome sequencing also showed no significant findings. After treatment with bromocriptine and short-acting contraceptive pills, the recurrent ovarian cysts resolved spontaneously, and hormonal levels returned to normal ranges. **Conclusions:** In women of childbearing age, it is important to conduct thorough endocrine evaluations and genetic screenings following the occurrence of large ovarian follicular cysts. Once endocrine levels are balanced, follicular cysts may decrease in size substantially, which helps to avoid unnecessary ovarian surgery.

## 1. Introduction

Ovarian cysts are a common condition in women, with various causes that can be either physiological or pathological. Pathological ovarian cysts usually require surgical intervention, while most physiological ovarian cysts, like follicular cysts, tend to resolve on their own without the need for special treatment [1].

Ovarian follicular cysts are benign cystic formations caused by the failure of follicular ovulation. These cysts are commonly seen in women of reproductive age and are typically small and asymptomatic. However, some women may experience pelvic discomfort or irregular menstrual cycles. Diagnosing ovarian follicular cysts is generally straightforward. These cysts arise due to various factors, including disruptions in the hypothalamic–pituitary–ovarian (HPO) axis, which can cause irregular LH levels, abnormal hormonal pulses, and frequencies. This results in continuous high estrogen and progesterone stimulation, leading to temporary or prolonged anovulation. As a result, the growing or atretic follicles retain fluid, causing cystic expansion. The cyst walls are lined with two layers of cells: granulosa cells on the inner layer and theca cells on the outer layer. Both layers can undergo luteinization, although cyst sizes typically do not exceed 8 cm [1].

As most follicular cysts are functional, they often disappear spontaneously within a few months. Diagnosis is usually made through ultrasound, which helps assess the cyst’s morphology. In rare cases, when a cyst is persistent, abnormally large, or leads to complications such as rupture or ovarian torsion, surgical intervention may be required [2]. Generally, follicular cysts resolve on their own. However, persistent cysts may raise suspicion of underlying conditions such as pituitary gonadotropinomas, iatrogenic ovarian hyperstimulation, hypothyroidism, aromatase deficiency, or neoplastic lesions like adult granulosa cell tumors [2,3].

Herein, we report on two biological sisters who both developed large ovarian follicular cysts, which recurred after surgical treatment.

## 2. Cases Presentation

### 2.1. Case 1

#### 2.1.1. Clinical Presentation

The younger of the two sisters, a 16-year-old girl, presented to our gynecology department at the Second Affiliated Hospital of Chongqing Medical University on 15 February 2023, with a palpable abdominal mass that had been present for 2 months. She reported mild chest tightness but had no symptoms such as headaches, nausea, vomiting, abdominal pain, or abnormal vaginal bleeding. She denied any history of sexual activity. She began menstruating at age 13, with a menstrual cycle of 60 days, a 10-day period, and a normal flow. Her personal and familial medical history was unremarkable.

On physical examination, her vital signs were stable, and her BMI was 22.86 kg/m^2^. There was no acne on her face, and her hair growth was normal. There was no Adam’s apple, her breasts had developed appropriately without lactation, but her abdomen was significantly distended. The abdominal wall was tense, but there was no tenderness. Genital examination revealed dense pubic hair and a thickened clitoris, approximately 2 cm in length.

#### 2.1.2. Diagnostic Workup

**Figure 1:**
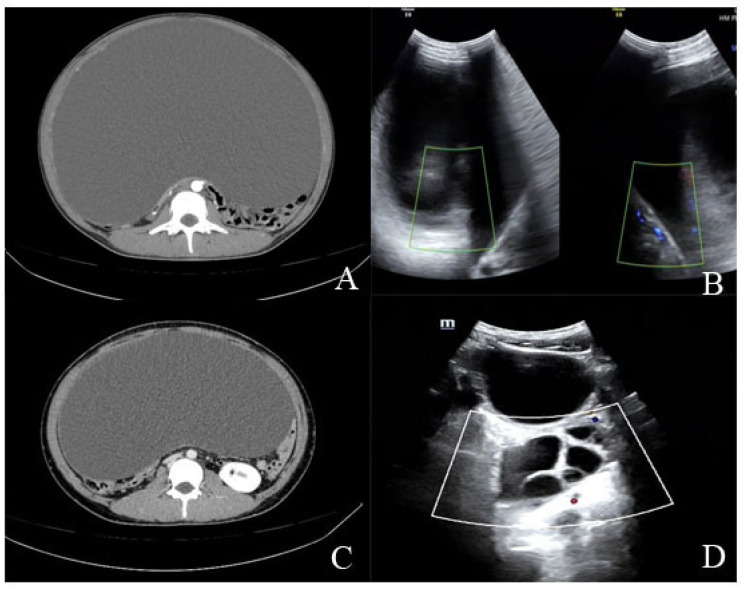
(**A**,**B**) Sister 1 preoperative whole-abdominal CT and color ultrasound. (**C**,**D**) Sister 2 preoperative whole-abdominal CT and color ultrasound.

Genetic testing was performed showing an XX sex chromosome pattern. Imaging examination, including a gynecological color Doppler ultrasound and abdominal CT scans, revealed a large cystic mass in the pelvic and abdominal cavities, with a maximum diameter of about 35 cm (Figure 1). Tumor markers, including cancer antigen 125 (CA125), human epididymis protein 4 (HE4), alpha-fetoprotein (AFP), and carcinoembryonic antigen (CEA), were all within normal ranges. Based on these findings, she was diagnosed with a large abdominal mass. The rest of the patient 1 diagnostic workup results are provided in Supplementary Materials.

#### 2.1.3. Treatment and Outcomes

**Figure 2:**
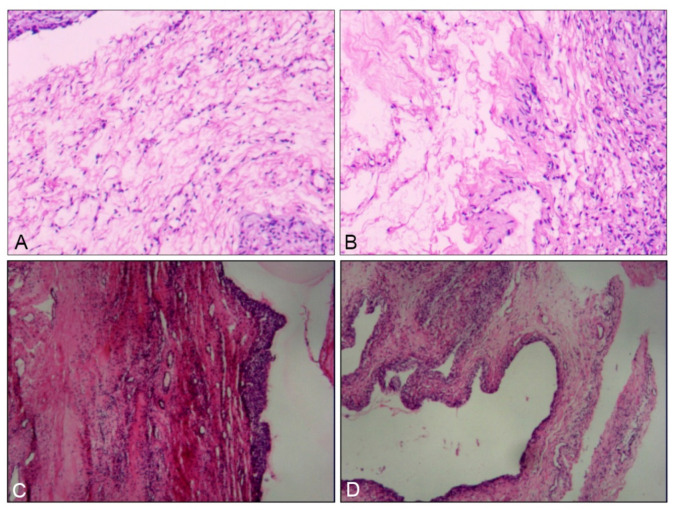
HE staining of the two sisters’ postoperative pathology. (**A**,**B**) Postoperative pathological images of the elder sister’s cyst. (**C**,**D**) Postoperative pathological images of the younger sister’s cyst.

On 17 February 2023, she underwent transabdominal exploration and bilateral ovarian cystectomy. Intraoperatively, a large cyst was found in the right ovary and an 8 cm cyst in the left ovary. Approximately 8000 mL of clear fluid was aspirated from both cysts, and the cyst walls were smooth. Pathological analysis confirmed a right ovarian serous cystadenoma and a left ovarian follicular cyst (Figure 2).

**Figure 3:**
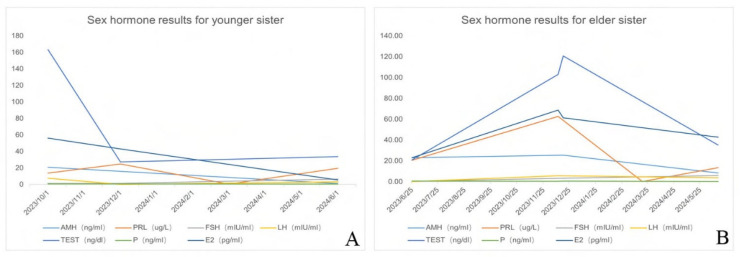
(**A**) Sex hormone results for sister 1. (**B**) Sex hormone results for sister 2.

Eight months after the operation, a follow-up Doppler ultrasound revealed cystic masses in the adnexal regions on both sides, measuring approximately 8 cm on the right and 7 cm on the left. Hormone levels were as follows: anti-Müllerian hormone (AMH) 20.77 ng/mL, estradiol (E2) 56.06 pg/mL, FSH 1.35 mIU/mL, LH 7.60 mIU/mL, prolactin (PRL) 13.77 µg/L, progesterone (P) 0.63 ng/mL, and testosterone 163.20 ng/dL (Figure 3). Thyroid function was normal. To manage the cysts, GnRH-agonist (leuprorelin 3.75 mg) was administered subcutaneously once every 28 days for a total of two injections.

Despite this, 10 months post-operation, the cysts had increased in size, measuring 12 cm on the right and 11 cm on the left. Hormonal assays showed E2 at 43.00 pg/mL, FSH at 1.12 mIU/mL, LH at less than 0.20 mIU/mL, PRL 24.77 µg/L, P 0.73 ng/mL, and testosterone 27.18 ng/dL. Other endocrine parameters, including dehydroepiandrosterone sulfate, ACTH, and cortisol, were normal. Family exome sequencing (including mother, father, sister, and the patient) revealed no abnormalities, and pituitary MRI was normal.

The patient was treated with oral ethinyl estradiol cyproterone acetate tablets and bromocriptine. Three months into this treatment (13 months post-surgery), the cysts had reduced in size to approximately 8 cm on both sides, and PRL levels returned to normal (0.39 µg/L). Bromocriptine was discontinued while the ethinyl estradiol cyproterone acetate treatment continued.

Sixteen months post-surgery, the cysts had further shrunk, with the right cyst measuring 4 × 2 cm, while the left cyst had disappeared. Additionally, the clitoral size reduced to about 1.0 cm. Hormonal levels had normalized: AMH 1.13 ng/mL, E2 5.50 pg/mL, FSH 6.58 mIU/mL, LH 3.06 mIU/mL, PRL 19.60 µg/L, P 0.42 ng/mL, testosterone 33.72 ng/dL. At the 18-month follow-up, the patient’s hormones remained normal, and there were no signs of cyst recurrence.

### 2.2. Case 2

#### 2.2.1. Clinical Presentation

The elder sister, aged 19, presented to the same hospital on 23 June 2023, with a 4-month history of a palpable abdominal mass. She experienced abdominal distension and constipation but denied other symptoms such as headache, nausea, vomiting, abdominal pain, or abnormal vaginal bleeding. She also denied any sexual history. Menarche occurred at age 13, with a menstrual cycle of 60 days and a 10-day period, which was normal in flow. There were no notable abnormalities in her medical, family, or personal history.

On physical examination, her vital signs were stable, with a BMI of 22.49 kg/m^2^. She had facial acne and dense body hair. Her throat had a slight prominence resembling an Adam’s apple, her breasts were developed with no lactation, and her abdomen was noticeably distended with high tension in the abdominal wall but no tenderness. Pubic hair was dense, and her clitoris was enlarged to about 2.5 cm.

#### 2.2.2. Diagnostic Workup

Imaging with pelvic and abdominal ultrasound and a CT scan showed a large cystic mass in the pelvic and abdominal cavity, with a maximum diameter of 43 cm (Figure 1). Tumor markers were normal. With her sister’s medical history in mind, preoperative lab tests revealed elevated AMH (>22.96 ng/mL), low FSH (0.32 mIU/mL), and low LH (<0.10 mIU/mL), alongside elevated testosterone levels (20.83 ng/dL). Other hormone levels were normal, and sex chromosomes were confirmed as XX. The rest of the patient 2 diagnostic workup results are provided in Supplementary Materials.

#### 2.2.3. Treatment and Outcomes

On 26 June 2023, she underwent transabdominal exploration and bilateral ovarian cystectomy. A large cyst originating from the right ovary and a smaller 11 cm cyst in the left ovary were drained, yielding 18,000 mL of fluid. A histopathology study revealed a right ovarian follicular cyst and a left ovarian mature teratoma (Figure 2).

Four months later, a 7 cm cyst was detected in the left ovary, and by six months post-surgery, masses of 5 cm and 7 cm were noted in the pelvic cavity. Hormone levels showed increased AMH (25.40 ng/mL) and testosterone (102.93 ng/dL), with a significantly elevated PRL level of 62.71 µg/L. Chromosome sequencing of family members showed no abnormalities, and pituitary MRI was normal.

Treatment with oral ethinyl estradiol cyproterone acetate tablets and bromocriptine led to a significant reduction in PRL to normal levels (0.21 µg/L), and by 9 months, the cysts had disappeared. After 12 months, there was no recurrence, and the clitoris had reduced to 1.5 cm. AMH decreased, and other sex hormones normalized (Figure 3). At the 18-month follow-up, hormone levels remained stable, and there was no recurrence of ovarian cysts.

#### 2.2.4. Follow-Up

In both cases, we sent the patients back to the hospital for follow-up every 6 months. In addition to color ultrasound, the patients’ liver and kidney functions will be checked at each follow-up. We have no plans to stop oral contraceptives for the time being. Hence, the symptoms of hyperandrogenism in the two cases were obvious. During the follow-up, we tried to stop the medication, but irregular menstruation would occur; therefore, oral contraceptives will be continued to prevent any recurrences.

## 3. Discussion

### 3.1. Case Analysis

The two sisters, born to the same parents, presented with notably similar medical histories. Their common features were as follows: (i) Adolescent Girls of Childbearing Age with Irregular Menstruation: Both sisters experienced irregular menstrual cycles. (ii) Hyperandrogenic Signs: They showed external genitalia signs of hyperandrogenism-ism, including thickened clitorises. (iii) Presence of Giant Ovarian Cysts: Both had large ovarian cysts in the pelvic and abdominal cavities. One of the bilateral ovarian cysts was diagnosed as an ovarian follicular cyst, and the cysts recurred after surgery. (iv) Hormonal Imbalance: Both had elevated levels of AMH, PRL, and testosterone, with low levels of FSH and LH. (v) Positive Response to Treatment: Both responded effectively to treatment with oral short-acting contraceptives and the dopamine analogue bromocriptine (Table 1).

Following surgeries, we discussed with the pathologists the masses’ nature. They believed that the structure of the follicular cyst was relatively simple and the diagnosis was very clear. There was no need to perform immunohistochemistry. Later, we consulted higher-level pathologists and obtained the same results.

The family history revealed no ovarian cysts in the grandmother or mother, and no mutations related to their clinical presentation were found through whole exome sequencing of the family, covering the exon regions of approximately 20,000 genes in the human genome, including the mitochondrial genome. From a genetic point of view, the fact that family exome sequencing revealed no significant findings reduces if not excludes the chance of the heritable nature of the underlying genetic defects leading to the observed susceptibility to ovarian cysts formation and recurrence. We can argue that the above results suggest the potential acquisition of the genetic anomalies during the pre and/or post-natal life while their co-occurrence in the two sisters could result from the exposure to similar environmental factors perhaps leading to epigenetic modifications (e.g., DNA methylation, histone modifications) affecting genes within the gonadal and/or extragonadal tissue. Nevertheless, there is a difference between inherited genetic mutations and genetic polymorphism as the latter is unlikely to be detected by whole exome sequencing. Special polymorphisms in families are associated with the development of ovary cysts causing diseases such as Polycystic Ovary Syndrome (PCOS), and contribute to familial susceptibilities [4]. The great similarity in the clinical, biological, morphological, and pathological aspects of the ovarian cysts’ presentation as well as their reoccurrence and treatment response support the development of similar aberrant genetic modifications among the sisters. Novel acquired heterozygous mutations can also develop and contribute to ovary cysts in sisters without necessarily being detected in exome sequencing of the mother/grandmother [5].

Complete surgical removal of the ovarian masses was presumably achieved during the primary intervention due to extensive masses resections. Therefore, it is likely that the reoccurrence was attributed to the persistence of an etiological factor maintaining in-creased levels of androgens (e.g., PCOS, pituitary gonadotropins secreting tumor, or androgen-producing tumor) with hyperstimulating/disrupting effects on the ovarian cells’ growth. This would have favored de novo formation of the ovary cysts. Thus, mechanistically elevated AMH and testosterone can contribute to the development of cystic masses by inhibiting the maturation of the ovarian follicles favoring cystic transformation [6,7]. These follicular cystogenesis-inducing effects would have been neutralized by the oral contraceptives therapy as the latter reduces the action of androgens (both AMH and testosterone) [8,9].

### 3.2. Analysis of the Causes of Recurrent Ovarian Cysts

Both sisters underwent surgery for giant ovarian cysts, with postoperative pathology confirming ovarian follicular cysts. However, the cysts recurred shortly after surgery. To preserve ovarian function and minimize harm, further surgery was not immediately pursued. The exact cause of the recurrent ovarian cysts remains unclear, and similar cases are scarcely reported in the literature. Based on their hormonal profiles, showing low gonadotropins and high AMH, as well as their examination results and relevant literature, several potential causes of cyst recurrence have been considered from the least probable to the most probable.

#### 3.2.1. Cystic Granulosa Cell Tumors

Diagnosing granulosa cell tumors, originating from ovarian sex cord stromal tissue, we can see that they have low malignant potential. It can be difficult to distinguish these from ovarian follicular cysts, particularly in cases where the cyst exceeds 8 cm or if the patient presents with early puberty or virilizing features. In such scenarios, distinguishing between follicular cysts and cystic granulosa cell tumors, including the adult and juvenile types, is crucial [10]. However, our patients’ pathological findings were not in favor of granulosa cell tumors.

#### 3.2.2. Aromatase Deficiency (AD) and Ovarian Luteinized Cysts

Aromatase deficiency (AD) is a rare autosomal recessive genetic disorder, with only a limited number of cases reported in the literature. AD results from mutations in the CYP19 gene, which encodes the enzyme aromatase (P450arom). Aromatase is responsible for converting androgens into estrogens, and is expressed in various tissues, including ovarian granulosa cells, luteal cells, testes, adipose tissue, liver, and hypothalamus. This enzyme is crucial for estrogen synthesis, reproduction, and metabolic processes. In patients with AD, the inability to convert androgens into estrogen leads to estrogen deficiency and androgen excess. As a result, affected individuals may exhibit clitoral enlargement, underdeveloped breasts, ovarian hyperstimulation, and polycystic ovaries [11,12]. Treatment with estrogen replacement therapy can help resolve ovarian cysts, promote breast development, and restore normal menstruation [13].

Both sisters showed hyperandrogenism signs and short-acting contraceptives were effective, but genetic testing through whole exome sequencing did not reveal any variants related to AD or its clinical phenotype. While both PCOS and AD can present with hyperandrogenism and cystic ovaries, AD typically presents with low estrogen levels, which were not observed in the reported cases. Therefore, the diagnosis of AD is much less probable.

#### 3.2.3. Hyperprolactinemia and Polycystic Ovarian Changes

Hyperprolactinemia has been implicated as a possible cause of ovarian enlargement and polycystic ovarian morphology. In hypothyroidism, prolonged feedback mechanisms can cause an increase in thyrotropin-releasing hormone (TRH) from the hypothalamus, which stimulates the production of PRL [14]. High PRL levels suppress the pituitary–gonadal axis, inhibit LH secretion, and increase FSH sensitivity, which promotes follicular development [15].

Studies have also shown that elevated PRL levels are associated with the formation of ovarian cysts in animal models, and treatment with bromocriptine—a dopamine agonist—can reduce PRL levels and inhibit cyst formation [16]. The sisters had hyperprolactinemia but no thyroid dysfunction, prolactinoma, or other apparent hyperprolactinemia-inducing etiology. Following treatment with bromocriptine, PRL levels normalized, and although the elder sister’s PRL levels increased after discontinuing the medication, ovarian cysts did not recur. Together, these findings may indicate that hyperprolactinemia was not the primary etiopathogenic factor in ovarian cyst formation.

#### 3.2.4. Pituitary Gonadotropinoma and Ovarian Hyperstimulation Syndrome

A functional pituitary gonadotropinoma can cause ovarian hyperstimulation syndrome (OHSS), which requires the secretion of biologically active FSH. High serum FSH levels stimulate the ovaries to increase estrogen production. This, in turn, feeds back to the hypothalamus and pituitary, resulting in elevated PRL and suppressed LH. In adolescent girls, this may manifest as irregular menstruation, abnormal breast development, and abdominal distension due to ovarian enlargement.

Approximately 35% of pituitary gonadotropinomas can cause abnormal FSH/LH ratios in the serum, often leading to elevated FSH levels [17]. Surgical removal of the tumor is the most effective treatment, though drug therapies are typically used when surgery is not feasible or is unsuccessful [18]. Recurrence of ovarian cysts after stopping drug treatment has been associated with pituitary gonadotropinomas [3].

A notable case in the literature describes a patient who developed OHSS after treatment with GnRH-agonists [19]. This patient experienced bilateral ovarian enlargement, with hormone profiles showing an abnormal LH/FSH ratio, low LH and FSH, elevated PRL, and normal estradiol E2. Despite the cyst continuing to grow, no pituitary adenoma was detected on the MRI. The clinical and hormonal presentations were similar to pituitary gonadotropinoma, but no tumor was identified in the pituitary. This case underscores the complexity of diagnosing pituitary-related ovarian cysts.

Our patients’ brain MRI did not reveal any pituitary adenoma, which does not necessarily indicate the absence of focal gonadotropinoma or other pituitary proliferations, as microadenomas, even when clinically impactful, can be non-visible on an MRI [20]. To the best of our knowledge, a paraneoplastic section of gonadotropins has been reported. Despite these arguments, the low levels of FSH and LH, and the overall context, are less suggestive of gonadotropinoma. For instance, these already rare tumors are clinically silent in the overwhelming majority of cases, and are uncommon under the age of 25 years [21]. It is also exceptional that two sisters would develop a pituitary tumor that remains invisible on the MRI in both cases.

#### 3.2.5. Polycystic Ovary Syndrome (PCOS)

The primary clinical features of PCOS include hyperandrogenism, ovulatory dysfunction, and polycystic ovarian morphology. It is the most common endocrine disorder among women of reproductive age and has significant effects on reproductive, metabolic, and psychological health [22]. The diagnosis of PCOS is typically based on the Rotterdam criteria, which require two of the following: clinical and/or biochemical signs of androgen excess, ovulatory dysfunction, and the presence of polycystic ovaries on ultrasound, often accompanied by elevated AMH levels [23].

There is no definitive threshold for AMH in PCOS diagnosis, though serum AMH levels in PCOS patients are typically two to three times higher than those seen in women with normal ovarian function [24,25]. While no universal standard exists for ovarian volume in PCOS diagnosis via ultrasound, studies suggest a critical range of 6–13 cm^3^ [26].

First-line treatment for both adult and adolescent PCOS patients, especially when a clear diagnosis is made, is short-acting contraceptives (COCs). Along with hormonal treatment, comprehensive management should include lifestyle interventions, weight management, and psychological evaluation [23,27].

In the present report, the two sisters exhibited elevated AMH, androgen excess, and hyperprolactinemia, with associated symptoms such as acne, hirsutism, and clitoral enlargement. Although COCs therapy proved effective, their ovarian volumes were unusually large, necessitating further clinical observation and monitoring, as their presentation may suggest atypical/incomplete PCOS cases. However, the diagnosis of PCOS may not be very surprising given the high prevalence (8–13% of reproductive-aged women [28]) and wide heterogeneity of this syndrome. Cases of sisters/twins with PCOS have been previously reported [5,29,30]. The occurrence of a giant ovarian cyst in the setting of PCOS has also been described in young-age patients [31,32]. Furthermore, in the first sister, the LH/FSH ratio was 7:1 (normally 1:1) while the second had an LH/FSH ratio < 1. Although typically there is an increase in gonadotropins, both increased and decreased LH/FSH ratios are suggestive of PCOS [33]. Hyperprolactinemia is common during PCOS, affecting 37% of patients and can be secondary (e.g., due to pituitary adenoma) or idiopathic (non-organic) [34]. Besides restoring menstrual cyclicity and improving hyperandrogenism, oral contraceptives are effective in decreasing the size of the ovarian cysts [35,36].

Although the etiological diseases of these two sisters were not clear, we thought that by improving the endocrine status, the recurrent cysts would become smaller and disappear. Fortunately, this was the case. This may remind physicians that when dealing with recurrent follicular cysts with unclear etiology, after ruling out other diseases, they should pay attention to the endocrine status and attempt to correct any hormonal imbalance if possible. In case of failure, cautiously performing conservative surgeries to protect the patients’ reproductive function is the reasonable approach. Future reports on rare ovarian follicular cyst presentations are needed as they may help establish a better understanding of this condition’s heterogeneity, hence optimizing diagnosis and management approaches.

## 4. Conclusions

In summary, these two sisters shared highly similar clinical presentations, including recurrent giant ovarian cysts, hyperandrogenism, and abnormal LH/FSH ratios. Despite no known family history of genetic disease, no special dietary habits, and no particular medications, the same treatment plan based on oral contraceptives was effective for both patients avoiding iterative surgeries. However, the underlying cause remains unclear, requiring further investigation. Diagnoses such as granulosa cell tumors, AD and gonadotropinoma appear to be less probable, while an atypical PCOS seems to have a higher possibility. Sharing this case could offer valuable insights into the diagnosis and treatment of recurrent ovarian cysts and may help explore the potential causes of this rare condition.

## Figures and Tables

**Table 1:** Summary of the two sisters’ initial findings.

	Case 1 (Younger Sister)	Case 2 (Elder Sister)
Age (years)	16	19
BMI	22.86 kg/m^2^	22.49 kg/m^2^
Hyperandrogenism signs	Distended abdomen, dense pubic hair and a thickened clitoris	Facial acne, dense body hair, Adam’s apple-like prominence, distended, abdomen, dense pubic hair and enlarged clitoris
Imaging findings	Large ovarian cystic mass with a maximum diameter of about 35 cm	Large ovarian cystic mass with a maximum diameter of 43 cm
Ovarian tumor markers	Normal	Normal
Pelvic laparotomy findings	Large cyst in the right ovary and an 8 cm cyst in the left ovary	Large cyst in the right ovary and a smaller 11 cm cyst in the left ovary
Pathological diagnoses	Right ovarian serous cystadenoma and a left ovarian follicular cyst	Right ovarian follicular cyst and a left ovarian mature teratoma
Hormonal anomalies	Increased AMH, testosterone, and prolactin Decreased LH and FSH	Increased AMH and testosterone Decreased LH and FSH
Recurrence of the ovarian cysts after surgery	Yes	Yes
Sustained response to contraceptives	Yes	Yes

## Data Availability

Data are contained within the article and Supplementary Materials.

## Supplementary Materials

The following supporting information can be downloaded at: https://www.mdpi.com/article/10.3390/healthcare13060656/s1.
