# Supplementary material for: Recurrent Giant Ovarian Cysts in Biological Sisters: 2 Case Reports and Literature Review—Giant Ovarian Cysts in 2 Sisters
Source: Healthcare (Basel). 2025 Mar 17;13(6):656. doi: 10.3390/healthcare13060656 (PMC11942049; doi:10.3390/healthcare13060656)
Supplement: Supplementary file 1 [file healthcare-13-00656-s001.zip › healthcare-3452529-supplementary.pdf]

Recurrent Giant Ovarian Cysts in Biological Sisters: 2 Case

Reports and Literature Review

Supplementary material

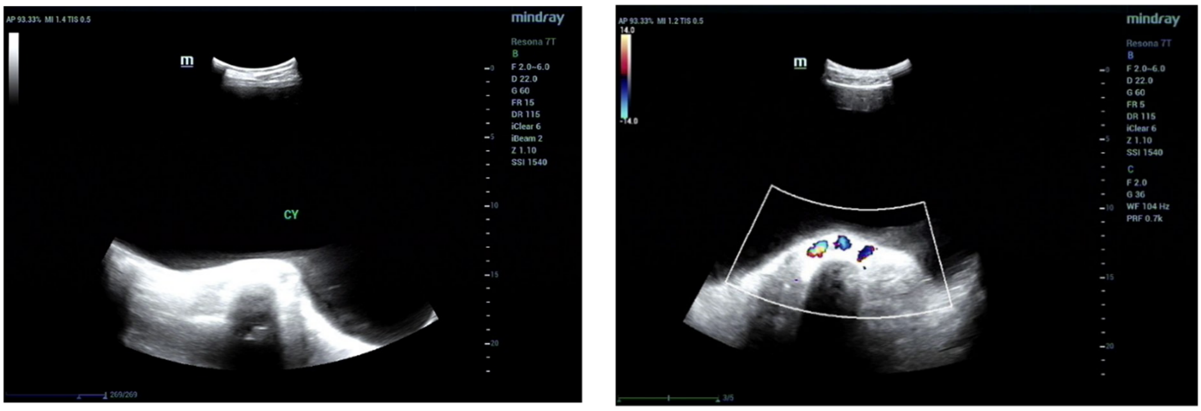

Case1 Color Doppler Ultrasound at 2023-02-15 (at presentation).

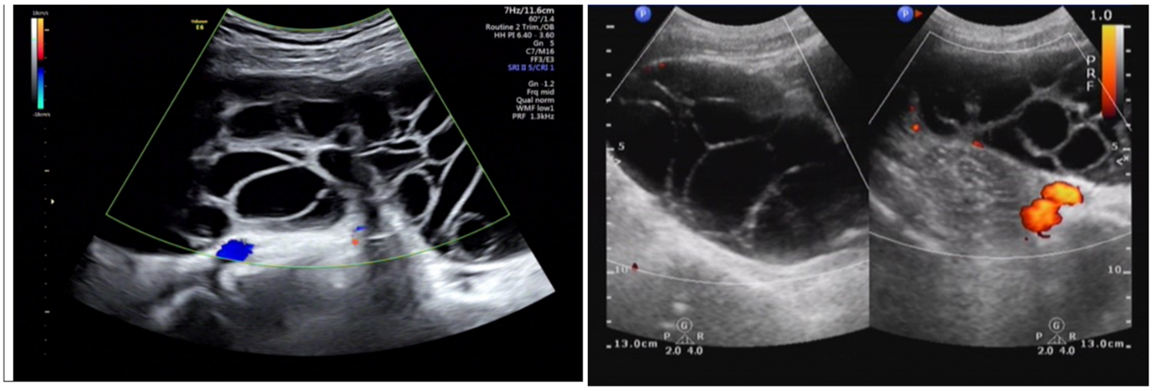

Case1 Color Doppler Ultrasound at 2023-12-11 (after recurrence).

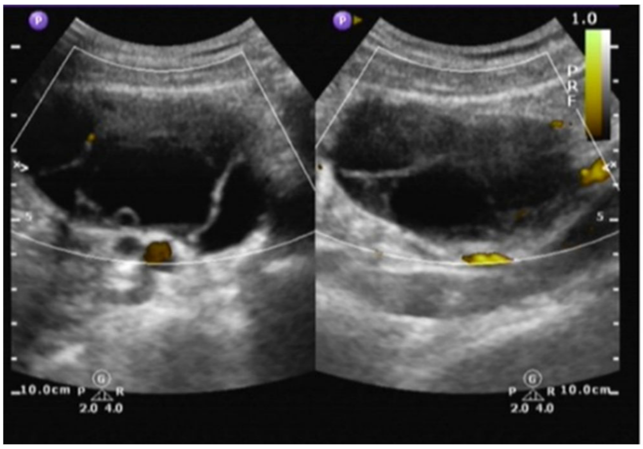

Case1 Color Doppler Ultrasound at 2024-3-19 (during treatment with contraceptives)

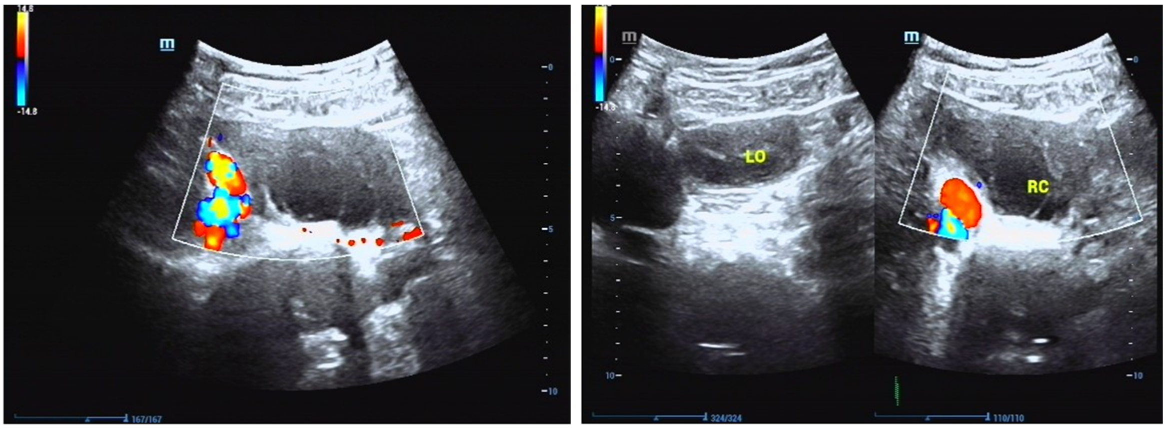

Case1 Color Doppler Ultrasound at 2024-6-15 (during post ovarian cysts regression follow-up).

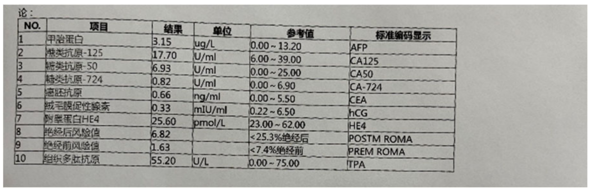
论：

NO.	项目	结果	单位	参考值	标准编码显示
1	甲胎蛋白	3.15	ug/L	0.00～13.20	AFP
2	糖类抗原-125	17.70	U/ml	6.00～39.00	CA125
3	糖类抗原-50	6.93	U/ml	0.00～25.00	CA50
4	糖类抗原-724	0.82	U/ml	0.00～6.90	CA-724
5	癌胚抗原	0.66	ng/ml	0.00～5.50	CEA
6	绒毛膜促性腺素	0.33	mIU/ml	0.22～6.50	hCG
7	附睾蛋白HE4	25.60	pmol/L	23.00～62.00	HE4
8	绝经后风险值	6.82		<25.3%绝经后	POSTM ROMA
9	绝经前风险值	1.63		<7.4%绝经前	PREM ROMA
10	组织多肽抗原	55.20	U/L	0.00～75.00	TPA

Results of ovarian cancer markers for sister 1 indicating levels within normal range.

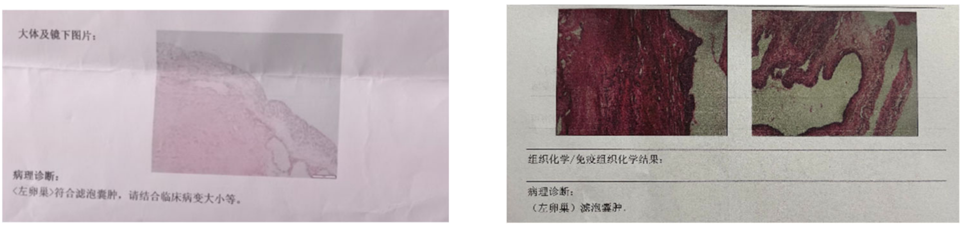

Case 1 pathological findings from Chongqing Medical University affirming the diagnosis of ovarian follicular cysts. ***Left figure:*** First pathological report indicating the diagnosis of left ovary consistent with follicular cyst. *(Address: Pathology Department, First and Second Floors, Shangyi Building, Chongqing Medical University Campus. Report Date: 2023-12-14; 023-68485789*); ***Right figure:*** Second pathological report indicating the diagnosis of left ovary cyst *(Reporting Physician: Zhu Yunzhe, Reviewing Physician: Gu Xiling; Xiaolu 18m: #19 13800824142, Examination Date: 2023-02-20, Report Date: 2023-02-21 08:53:22; Contact number: (023) 62887425).*

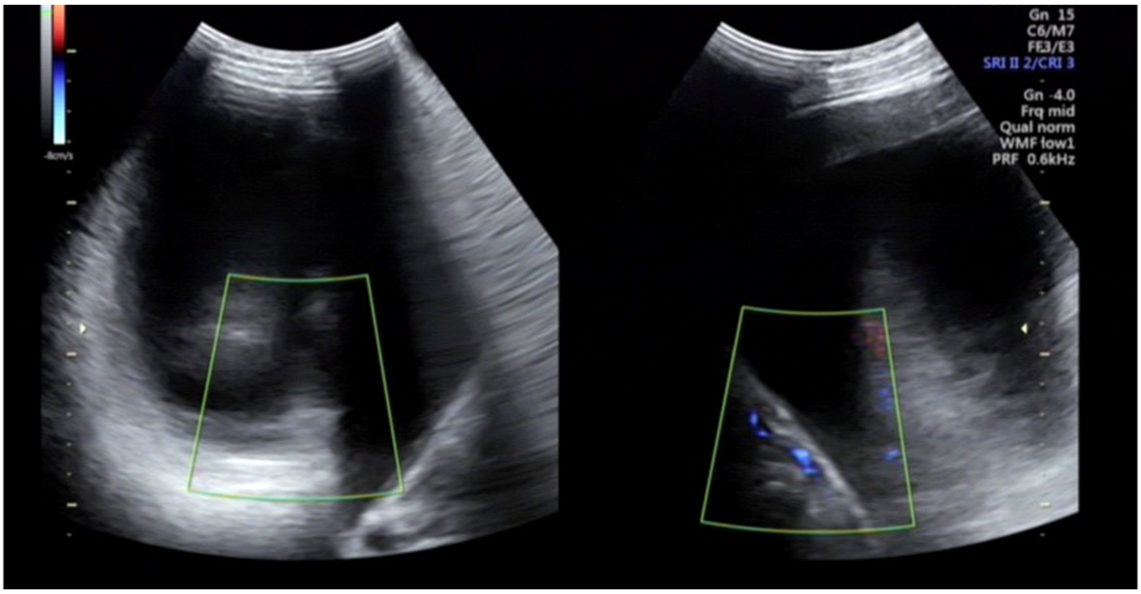

Case 2 Color Doppler Ultrasound at the presentation (2023-6-23).

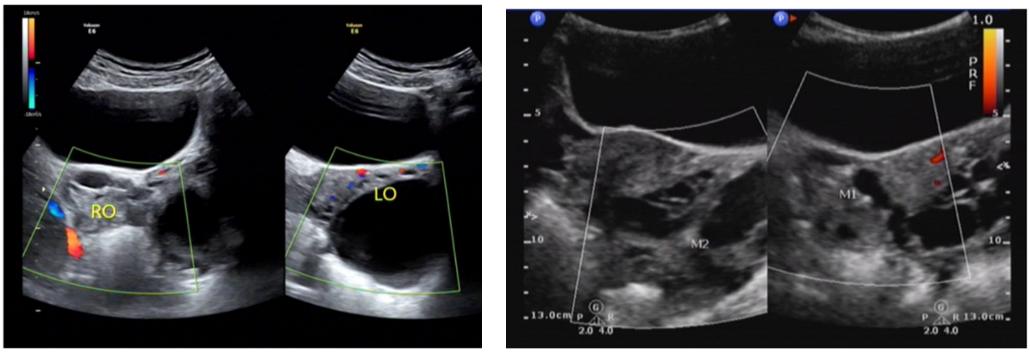

Case 2 Color Doppler Ultrasound after recurrence.

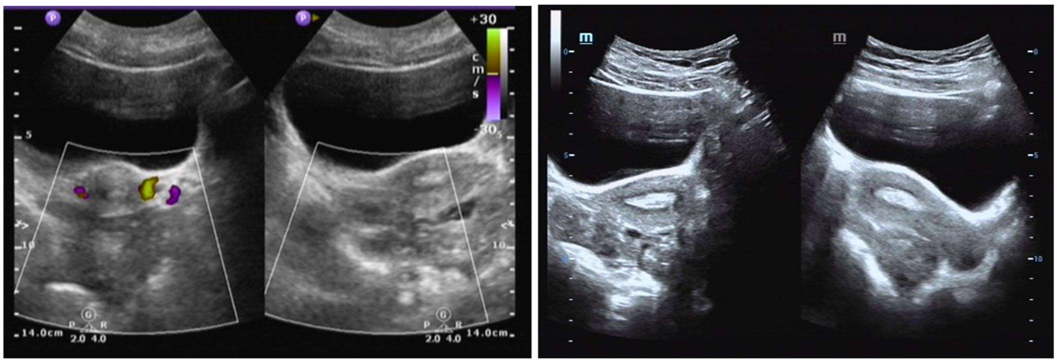

Case 2 Color Doppler Ultrasound during treatment with contraceptives.

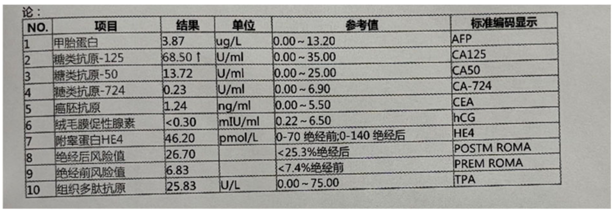

论：

NO.	项目	结果	单位	参考值	标准编码显示
1	甲胎蛋白	3.87	ug/L	0.00～13.20	AFP
2	糖类抗原-125	68.50 ↑	U/ml	0.00～35.00	CA125
3	糖类抗原-50	13.72	U/ml	0.00～25.00	CA50
4	糖类抗原-724	0.23	U/ml	0.00～6.90	CA-724
5	癌胚抗原	1.24	ng/ml	0.00～5.50	CEA
6	绒毛膜促性腺素	<0.30	mIU/ml	0.22～6.50	hCG
7	附睾蛋白HE4	46.20	pmol/L	0-70 绝经前;0-140 绝经后	HE4
8	绝经后风险值	26.70		<25.3%绝经后	POSTM ROMA
9	绝经前风险值	6.83		<7.4%绝经前	PREM ROMA
10	组织多肽抗原	25.83	U/L	0.00～75.00	TPA

Results of ovarian cancer markers for sister 2 indicating levels within normal ranges.

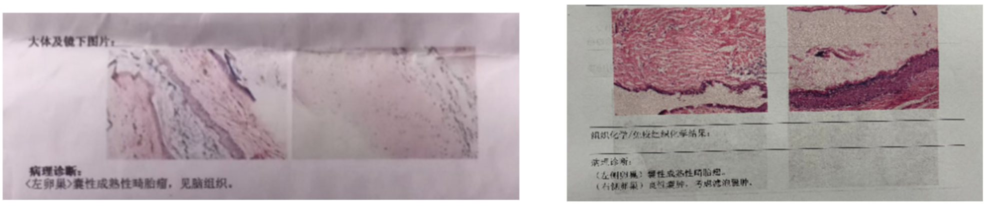

Case 2 pathological findings from Chongqing Medical University affirming the diagnosis of ovarian follicular cysts. First (left figure) and second (right figure) pathological reports indicating the diagnosis of cystic left ovary and mature teratoma, with presence of brain tissue (*Reporting Doctor: Ye Xiufeng, Guan Guanxiao Qin; 100368, Report Date: 2023-12-14, Phone: 023-89012223 023-68485789, Address: First Floor, Shangi Building, Second Floor Pathology Department, Chongqing Medical University*).
